# Contrast-Enhanced Mammography-Guided Biopsy in Patients with Extensive Suspicious Microcalcifications

**DOI:** 10.3390/cancers17183086

**Published:** 2025-09-22

**Authors:** Yun-Chung Cheung, Wai-Shan Chung, Ya-Chun Tang, Chia-Wei Li

**Affiliations:** 1 Department of Medical Imaging and Intervention, Chang Gung Memorial Hospital, Medical College, Chang Gung University, Taoyuan 33382, Taiwan; 2 Division of Breast Surgery, Department of Surgery, Chang Gung Memorial Hospital, Medical College, Chang Gung University, Taoyuan 33305, Taiwan; 3 Taiwan Research Group, GE HealthCare, Taipei 10480, Taiwan

**Keywords:** contrast-enhanced mammography, contrast-enhanced mammography guided biopsy, breast microcalcifications, breast cancer, mammography, diagnosis

## Abstract

**Objectives**: To investigate the feasibility of contrast-enhanced mammography-guided biopsy (CEM-Bx) to diagnose cancer via targeting the associated enhancements in the patients with extensive suspicious microcalcifications. **Methods**: All the women with extensive suspicious microcalcifications were mammographically screened. Contrast-enhanced mammography was first examined, followed by CEM-Bx if there was any relevant enhancement; otherwise, patients without enhancement were submitted to conventional mammography-guided biopsy (MG-Bx). We recorded and analyzed the histological results, morphologies and distributions of the microcalcifications. The outcomes were also compared to those patients (control group) who did not assess with CEM and received MG-Bx only by the Wilcoxon rank-sum test. **Results**: Between November 2021 and November 2023, a total of 61 participants participated in the test. A total of 26 women underwent CEM-Bx, and 35 underwent MG-Bx. In total, 19 of the 26 CEM-Bx were diagnosed as cancer, and none by MG-Bx. The cancer diagnostic rates (CDRs) identified by CEM-Bx were 81.8% for regional microcalcifications and 66.7% for segmental or diffuse distributions. The CDR of the test group was higher than the control group, 31.4% to 20%, respectively. Otherwise, the CDR of CEM-Bx was significantly higher than MG-Bx in the control group (73.08% to 20%, *p*-valve < 0.01). **Conclusions**: CEM-Bx was a safe and feasible procedure. With identification of the enhanced target, CEM-Bx faithfully performed among the extensive distributed suspicious microcalcifications. Although CEM-Bx improves CDR, larger prospective trials with surgical validation of all lesions are needed before widespread adoption.

## Simple Summary

Contrast-enhanced mammography-guided biopsy (CEM-Bx) is a new technique that was approved for clinical use by the United States Food and Drug Administration in 2020. We designed this study to assess the performance of CEM-Bx in the screened women with extensive suspicious microcalcifications. According to the management strategy, such suspicious microcalcifications with cancer probability are essentially recommended mammography-guided biopsy (MG-Bx). For a focal needle sampling obtaining limit amount breast tissues, the cancer diagnosis depends on the correct target selection that is often confused among the extensive suspicious microcalcifications. By displaying the relevant enhancement, CEM-Bx was safe and feasible to obtain histologic diagnosis, resulting in a high cancer rate in this study.

## 1. Introduction

Breast cancer is the most common fatal disease of the female population worldwide, accounting for 24.2% of all cancers [1]. The five-year relative survival rate can increase to a range of 90.3–99% when breast cancer is detected early as a localized disease [2]. Breast mammographic screening has been recognized as the best way to detect early-stage breast cancer and reduce mortality from this disease [3,4,5,6]. In recent decades, the observed improvements in breast cancer survival have been attributed to the introduction of population-based mammographic screening [7]. The non-symptomatic cancers with only suspicious microcalcifications, particularly ductal carcinoma in situ (DCIS) or small invasive ductal cancer (IDC), can be discovered early, and approximately 20–30% were diagnosed by stereotactic mammography-guided biopsy (MG-Bx) [8,9,10,11]. The risk stratification of suspicious microcalcifications that were recommended for biopsy was basically assessed according to their mammographic morphology and distribution [12,13,14], and usually classified to category four, with malignant probabilities composing less than 2% of category 4a to over 95% of category 4c (<95%) [13].

MG-Bx is a well-established technique, promising to obtain suspicious microcalcifications for histological diagnosis. However, the problem we meet often is the confusion of target selection among the large area of suspicious microcalcifications, because all individual microcalcifications may be cancerous or non-cancerous. Currently, the modern technique of contrast-enhanced mammography (CEM) can reveal enhancements relevant to malignant microcalcifications [15,16] that can additionally localize the suspicious targets among the extensive microcalcifications. In this prospective study, we used contrast-enhanced mammography-guided biopsy (CEM-Bx) to biopsy the microcalcifications with relevant enhancements in patients with extensive suspicious microcalcifications that were believed helpful for target selection. To our knowledge, the clinical outcome of CEM-Bx to diagnose suspicious extensive microcalcifications has been investigated little in the literature.

## 2. Methods and Materials

### 2.1. Patient Selection

The study was approved by the institutional review board of Chang Gung Memorial Hospital (202102180A3), and the CEM-Bx project was supported by the National Science and Technology Council of Taiwan/Republic of China. Between November 2021 and November 2023, we performed CEM-Bx with vacuum-assisted biopsy needles in patients with extensive suspicious microcalcifications. The patients were recalled from the National Mammographic Screening Programme due to indeterminate microcalcifications. After finding the absence of associated mass, they were invited to join the project with explanation of the purpose and potential complications of CEM-Bx, including bleeding, infection and post-biopsy discomfort that were the same as the standard MG-Bx with vacuum-assisted biopsy needle, as well as the additional allergic reactions to iodinated contrast medium. After confirming normal renal function and coagulation blood tests, CEM was essentially performed days before biopsy (within one week of CEM-Bx) to determine whether there was relevant enhancement among the microcalcifications. The patients with relevant enhancement were subjected to CEM-Bx; otherwise, those without were subjected to MG-Bx.

Women who joined the CEM-Bx met all the inclusive criteria of (1) having extensive suspicious microcalcifications with BI-RADS 4 classification by mammographic screening; (2) having no associate mass found by physical examination, sonography and mammography; and (3) having relevant enhancement on CEM. The exclusive criteria included (1) allergic history to iodinated contrast medium; (2) renal function impairment; (3) pregnancy or lactation; or (4) hemostatic disorder. Herein, the enhancement associated to suspicious microcalcifications was defined as the presence of high attenuation on recombine enhanced images (REI) being concordantly over the suspicious microcalcification on the low-energy mammograms (LM). All the patients had signed consent forms according to our hospital’s clinical and research regulations.

**Figure 1:**
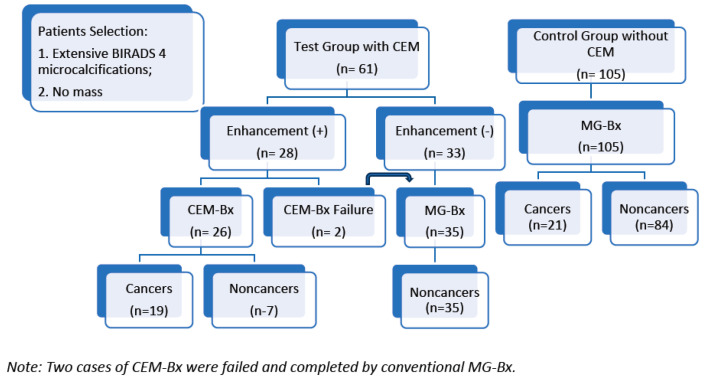
Diagnostic schema of the test and control groups.

To compare the cancer diagnostic ability of CEM-Bx to conventional MG-Bx (either by stereotactic or tomosynthesis guiding), we established a control group by reviewing the clinical data of patients with extensive suspicious microcalcifications. They were also recruited from the screened population without an associated mass during the same period of time. The patient selection flowchart is shown below (Figure 1).

In total, 61 women (age range: 46 years to 67 years) participated in the project. All the CEM and CEM-Bx were performed by the project investigator who has more than 10 years of experience in CEM reading and over 20 years in mammographic reading and biopsy. We used the Excel software (16.77) to record the mammographic findings for the microcalcification distribution and morphology, biopsy results and surgico-histological diagnoses. The microcalcifications were classified as amorphous, pleomorphic or linear/branched, and the distributions were classified as regional (>2 cm territory of microcalcifications in diameter), segmental (triangular territory with the tip pointing to the nipple) or diffuse, as well as whether associate enhancement present or not. The cancer diagnostic rate (CDR), upgrade of non-cancerous lesions to cancer, and upgrade of ductal carcinoma in situ (DCIS) were calculated. The results of the test and control groups were analyzed and compared, with the follow-up duration ranging from one to three years (mean of duration approximately two years).

### 2.2. Performance of CEM-Bx

CEM-Bx was conducted via a multifunctional mammographic machine (Pristina; GE Healthcare, Buc, France) that could perform digital mammography, tomosynthesis and contrast-enhanced mammography, as well as biopsy by means of stereotactic, tomosynthesis or contrast-enhanced imaging guided by an add-on biopsy system. For biopsy planning, we first evaluated the prior CEM to localize the target of suspicious enhancement. We also checked the compressed breast thickness for patient posture planning. For patients with breast thickness greater than 3 cm, the decubitus position with a vertical puncture direction (the needle direction perpendicular to compression plane) was preferred. If the breast thickness was 3 cm or less, the upright sitting position with a horizontal needle direction (the needle direction parallel to the compression plane) would be chosen. Repositioning of patient’s posture should essentially be avoided in which the delayed time might miss the optimal target enhancement. This was because the enhancement of the suspicious region and breast parenchyma started to dynamically change once the contrast medium was injected.

**Figure 2:**
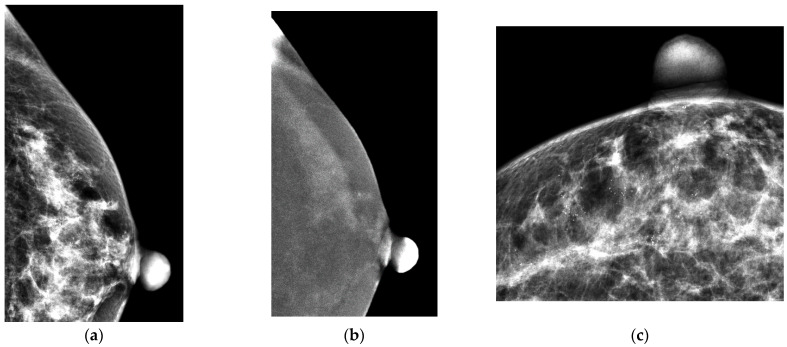

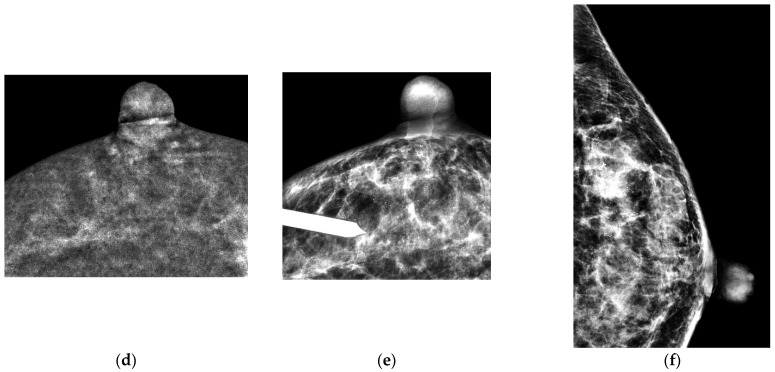
A 52-year-old woman with diffuse of amorphous and pleomorphous microcalcifications: The CEM-Bx and surgical results were both diagnosed to DCIS. (**a**) The LM (craniocaudal view) showed suspicious microcalcifications scattering in the outer region of left breast. (**b**) The REI (craniocaudal view) showed the presence of regional relevant enhancement in the outer region of left breast. (**c**) The pre-biopsy LM (craniocaudal view) localized the suspicious microcalcifications. (**d**) The pre-biopsy REI (craniocaudal view) revealed non-mass infiltrating enhancement. (**e**) The LM (craniocaudal view) showed the horizontal approach of biopsy needle beneath the target enhancement before firing the needle. (**f**) A ribbon-shape marker was placed at the biopsy site after CEM-Bx.

Before starting the procedure, the target was localized and marked with a red pen on the breast skin according to the last CEM. Coordination was measured by the perpendicular distances of enhancement to the skin and then to the nipple. The enhancement protocol for CEM-BX was the same as that for CEM with intravenous injection of contrast medium (Omnipaque 350 mg I/mL; GE Healthcare, Dublin, Ireland) at a rate of 3 mL/s (total dose of 1.5 mL/kg body weight). A total of 2 min after the injection of contrast medium, the marked target was placed and compressed at the center of the biopsy window. The stereotactic technique using the CEM exposure mode was then used to obtain a pair of LM and REIs at angles of 0, +15 and −15 degrees. Similarly to conventional stereotactic guided biopsy, we selected the enhanced target on the REIs, and the computerized coordination system of the machine guided the needle to the selected target. After confirming the correct position, the needle was fired through the target, and multidirectional suction samplings with complete clockwise rotation were then obtained by vacuum-assisted needle. Lastly, a micromarker was placed through the biopsy needle for localization. A case is demonstrated in Figure 2. Specimen mammography was routinely performed to determine whether microcalcifications were present within the specimens. After the control of local bleeding, mammography with craniocaudal and mediolateral obliques was performed to evaluate proper sampling as planned.

### 2.3. Data Analysis

The Shapiro—Wilk test was used to test the normality of all the datasets. Due to the nonnormality of all the data (*p* < 0.05), the Wilcoxon rank-sum test was used to examine the significance of between-group differences. The statistical software we used was R (version 4.5.1).

## 3. Results

### 3.1. Patient Samples of CEM-Bx

Twenty-eight participants with relevant enhancement were initially scheduled to undergo CEM-Bx. Among them, CEM-Bx was successful in twenty-eight patients, counting a 92.6% successful rate. The two failure cases were instantly completed by target selection by the pair of LMs in the same position, resembling the MG-Bx on the approximate microcalcifications. These two cases were thus attributed to the performance of using MG-Bx in the data analysis. The twenty-six CEM-Bx procedures were performed smoothly on the relevant enhancement via a vertical needle approach with the patient in the decubitus position (*n* = 19, 73%) and a horizontal needle approach with the patient in the upright sitting position (*n* = 7, 26.9%). For the other thirty-five patients by MG-Bx, twenty-two were in the decubitus position, with a vertical needle approach, and thirteen were in the upright sitting position, with a horizontal needle approach. The mean procedure time for CEM-Bx was 16.8 min (range, 7–23 min). The target selection was based on the performer’s decision among the suspicious microcalcifications. There were no severe complications and the specimen mammograms showed the presence of microcalcifications in all cases.

Although we followed pre-biopsy preparation protocols, two patients (7.1%) who were ready for CEM-Bx in our series were affirmably canceled. Both patients were performed in the decubitus position with vertical needle approach. The failure reason was because of poor identification of relevant enhancements. The failure causes were not understood; in fact, we assumed the poor observation of relevant enhancements in both cases might be due to the open window of the biopsy paddle without flat-plane compression.

The morphologies and distributions of the test group are summarized in Table 1. There was no significant difference in the morphology or distribution between CEM-Bx and MG-Bx.

### 3.2. Histopathologic Results

Of the 26 CEM-Bx patients, 19 patients were diagnosed with cancers (4 IDC and 15 DCIS), and 7 were diagnosed with non-cancerous lesions (3 flat epithelial atypia, 1 sclerosing adenosis, 1 atypical lobular hyperplasia, 1 fibrocystic disease and 1 benign ductal calcification). The CDR of CEM-Bx was 73.08%. Individually, the CDRs in the regional distribution were 81.8%, 66.7% in the segmental distribution and 66.7% in the diffuse distribution. All the patients with biopsy-proven cancers subsequently underwent surgery, resulting in 12 DCIS and 7 IDC. Surgical histology revealed that 2 of 15 (13.3%) DCIS cases were upgraded to IDC. None of the non-cancerous lesions were operated in the follow-up period; therefore, the upgrade to cancer could not be assessed. There was no breast cancer diagnosed. The sensitivity and specificity of CEM-Bx was 100%.

All 35 patients with MG-Bx were diagnosed with non-cancerous lesions. The CDR was 0%. However, one patient with atypical ductal hyperplasia subsequently underwent surgical excision and was ultimately upgraded to DCIS. The other patients were subjected to follow-up intervals, and no cancer was diagnosed. The outcomes are summarized in Table 1. The overall CDR of the microcalcifications with relevant enhancement by CEM-Bx was significantly higher than those by MG-Bx.

### 3.3. Comparison to the Control Group

The control group consisted of 105 patients. The morphologies and distributions of the microcalcifications and the biopsy outcomes in the test and control groups are summarized and compared in Table 2. A total of 21 patients were diagnosed with cancers (18 with DCIS and 3 with IDC) and 84 with non-cancerous disease. After surgery, 5 of the 18 patients (27.8%) with biopsy-proven DCIS were upgraded to IDC. On the other hand, four cases of the non-cancerous B3 microcalcifications received surgical excision and were finally diagnosed with two atypical ductal hyperplasia, one papillary neoplasm and one sclerosing adenosis. The overall CDR of the control group was 20%, lower than the 31.1% of the test group. Otherwise, the test group was analyzed as having higher CDR individually among the microcalcification distributions (regional, segmental, diffuse) and morphologies (coarse, amorphous, pleomorphous and linear) than the control group (Table 2). However, they were not statistically significant.

Importantly, the CDR of CEM-Bx was significantly higher than the control group (73.08% to 20%, *p*-value < 0.01). The DCIS upgrade rate of CEM-Bx was 13.3%, which was also lower than the 27.7% of the control group. The outcomes and CDR of the test and control groups are summarized in Table 2.

## 4. Discussion

CEM-Bx is a new technique that was approved by the United States Food and Drug Administration for clinical use in 2020 [17]. Alcantara et al. first reported the outcomes of a preclinical trial on CEM-Bx and reported the feasibility of this technique for diagnosing enhanced lesions on CEM [18]. The clinical application was still under investigation [19,20]. However, a few reports have proposed the use of CEM-Bx as an alternative to contrast-enhanced magnetic resonance imaging (CE-MRI)-guided biopsy [21,22], which can be considered in cases where CEM can display the same suspicion. The benefits of using CEM-Bx rather than CE-MRI can be easily explained by its lower cost, better machine availability, shorter procedural time and greater practicality for breast radiologists, which support the clinical applications of CEM-Bx. The advantages also exist between the examinations of CEM and enhanced MRI. Nevertheless, CEM-Bx is an option for suspicious lesions observed on CEM and is exclusive for the cases by CEM only.

CEMs have been shown to have greater cancer sensitivity than conventional mammography, tomosynthesis and sonography, and are approximate to CE-MRI [23,24,25]. Similarly to CE-MRI, CEM is a morpho-functional method under the same theory of cancer neoangiogenesis. However, it was interesting that targeted second-look sonography can morphologically identify only 31% of CEM-detected lesions [26]. The implementation of the technique of CEM-Bx is therefore essentially fulfilling the diagnostic gap left by CEM.

Regarding to the question of ‘why, when and where we need it’ of using CEM-Bx [27], in this study we tested the application of CEM-Bx in patients with extensive suspicious microcalcifications. When suspicious microcalcifications reveal associate enhancement, the cancer relevancy is greater than those without enhancement [15,16,28]. Based on cancer angiogenesis, the presence of relevant enhancement potentially indicates the likelihood of malignancy. A meta-analysis of 1843 lesions from 20 CE-MRI studies reported a pooled sensitivity and specificity of 92% and 82%, respectively, for BI-RADS 4 microcalcifications [29]. For CEM, the sensitivity and specificity have been reported to be 90.9% and 83.78% [15]. Otherwise, CEM is more easily observable and reliable for correlating microcalcifications on the LM to enhancements on the REIs in the same positioning session. An investigation recently reported a significantly greater PPV and lower misdiagnosis rate when using REIs than when using LMs alone [16]. That is the reason of why we compared the result of CEM-Bx to MG-Bx.

MG-Bx is commonly used to histologically identify the etiology of suspicious microcalcifications. The target is straightforward in the cases of grouped patterns. However, it is controversy when the distributions extend in regions, segment or diffuse. A recent paper reported that the overall CDR of MG-Bx was 20%. Furthermore, the CDRs were counted at 21% for grouped microcalcifications, 20% for regional or diffuse microcalcifications and 50% for segmental microcalcifications [30]. In our study, the CDRs of regional, segmental and diffuse microcalcifications of CEM-Bx were, respectively, 81.7%, 66.7% and 66.7%, which are comparatively higher than MG-Bx in our control group (18.2%, 25.9% and 0%, respectively). It might imply that the CEM with guiding biopsy helps to improve cancer diagnosis.

There were several limitations in this study. (1) The number of patients who were slated to receive CEM-Bx became smaller after evaluation by CEM. For those cases without relevant enhancement, CEM-Bx was not needed. In fact, we could not prospectively control the incidence of microcalcifications with relevant enhancements that definitely influence the sample size. Larger samples of CEM-Bx will be collected in the future. (2) The CDR was mainly assessed in this study. A lack of the surgery, sensitivity and specificity of the biopsy were virtually dependent on the follow-up. Actually, the interval mammographic follow-up is still essentially required for management. (3) The biopsy results of CEM-Bx and MG-Bx could not be subjected to case-to-case or lesion-to-case comparisons. In fact, each lesion could not receive biopsy twice by two different biopsy methods. We understood that there would be a bias for comparing CEM-Bx and MG-Bx in the test group. We therefore set a control from the clinical patients who received MG-Bx without CEM examination. The test and control groups were retrospective and derived from the cohort clinical data in the same period of time, not randomized. The bias might still be present.

## 5. Conclusions

CEM-Bx was a safe and feasible procedure. With identification of the enhanced target, CEM-Bx can be faithfully performed among the extensive distributed suspicious microcalcifications. Although CEM-Bx improves CDR, larger prospective trials with surgical validation of all lesions are needed before widespread adoption.

## Figures and Tables

**Table 1:** Appearances of microcalcifications and biopsy outcomes of the test group. The *p*-values showed the significance between the groups with and without the CEM-Bx process.

	CEM-Bx (*n* = 26)	MG-Bx (*n* = 35)	*p*-Value
Appearances			
Distribution			
Regional	11	14	0.9876
Segmental	9	11	
Diffuse	6	10	
Morphology			
Punctate	0	0	0.093
Amorphous	10	22	
Pleomorphous	15	12	
Linear	1	1	
Biopsy outcomes			
Cancers			
DCIS	15	0	<0.01
IDC	3	0	0.0181
ILC	1	0	0.2597
Noncancers			
ADH	0	1	0.4073
FEA	3	14	0.0154
ALH	1	2	0.8500
Adenosis	1	11	0.0081
Papillary neoplasm	0	1	0.4073
Fibrocystic	1	1	1.0000
Benign calcifications	1	5	0.1130
CDR % (*n*)	73.08 (19)	0	<0.01
DCIS upgrade rate % (*n*)	13.3 (2)	NA	0.4626

CDR = cases of cancer/total cases; DCIS = ductal carcinoma in situ; IDC = invasive ductal carcinoma; ILC = invasive lobular carcinoma; ADH = atypical ductal hyperplasia; FEA = flat epithelial atypia; ALH = atypical lobular hyperplasia; NA = not applicable. DCIS upgrade rate = cases of DCIS upgrade to IDC after surgery/cases of biopsy-proven DCIS.

**Table 2:** Biopsy outcomes of the test group and the control group.

Bx Diagnosis	Test (*n* = 61)	Control (*n* = 105)	*p*-Value
Cancers			
DCIS	15	18	0.0499
IDC	3	3	0.8025
ILC	1	0	0.4541
Noncancers			
ADH	1	6	1.0000
FEA	17	24	0.1816
ALH	3	1	0.2206
Adenosis	12	13	0.0842
Papillary neoplasm	1	1	0.6241
Fibrocystic	2	4	0.5883
Benign calcification	6	9	0.8404
Fibroadenoma	0	4	0.1547
Apocrine metaplasia	0	1	0.4900
Nonproliferation	0	1	0.4900
Columnar cell change	0	12	0.0105
Benign calcifications	0	8	0.0401
CDR			
Distribution			
Regional % (CA/Total)	36% (9/25)	19.2% (14/73)	0.0964
Segmental % (CA/Total)	30% (6/20)	25.9% (7/27)	0.6102
Diffuse % (CA/Total)	25% (4/16)	0% (0/5)	0.2493
Morphology			
Coarse % (CA/Total)	0	33.3% (1/3)	NA
Amorphous % (CA/Total)	21.8% (7/32)	17.2% (15/87)	0.2483
Pleomorphous % (CA/Total)	40.7% (11/27)	33.3% (5/15)	0.6510
Linear % (CA/Total)	50% (1/2)	0	NA
Overall CDR % (*n*)	31.14% (19/61)	20% (21/105)	0.0775
DCIS upgrade rate % (*n*)	13.32% (2/15)	27.7% (5/18)	0.4541

DCIS = ductal carcinoma in situ; IDC = invasive ductal carcinoma; ILC = invasive lobular carcinoma; ADH = atypical ductal hyperplasia; FEA = flat epithelial atypia; ALH = atypical lobular hyperplasia; NA = not applicable; cancer diagnostic rate (CDR) = % (cases of cancer/total cases); DCIS upgrade rate = cases of DCIS upgrade to IDC after surgery/cases of biopsy-proven DCIS; CA/Total = number of cancers/total cases in subcategory.

## Data Availability

The datasets generated or analyzed during the study are not publicly available since IRB approval is valid only for this research but are available from the corresponding author on reasonable request.

## Abbreviations

MG-Bx	mammography-guided biopsy
BI-RADS	Breast Imaging Reporting and Data System
DM	digital mammography
CEM	contrast-enhanced mammography
CEM-Bx	contrast-enhanced mammography-guided biopsy
LM	low-energy mammograms
REIs	recombine enhanced images
CDR	cancer diagnostic rate
DCIS	ductal carcinoma in situ
IDC	invasive ductal carcinoma
PPV	positive predictive value
CE-MRI	contrast-enhanced magnetic resonance imaging
